# Three-Arm Versus Four-Arm Configurations in Robot-Assisted Partial Nephrectomy: A Systematic Review and Meta-Analysis

**DOI:** 10.3390/jcm15031222

**Published:** 2026-02-04

**Authors:** Mohamed Javid Raja Iyub, Pushan Prabhakar, Deerush Kannan Sakthivel, Jasmine Pelia, Vivek Sanker, Manuel Ozambela Jr, Murugesan Manoharan

**Affiliations:** 1 Miami Cancer Institute, Baptist Health South Florida, Miami, FL 33176, USA; 2 Department of Neurosurgery, Stanford University, Stanford, CA 94305, USA; 3 Herbert Wertheim College of Medicine, Florida International University, Miami, FL 33199, USA

**Keywords:** robot-assisted partial nephrectomy, renal cell carcinoma, robotic surgery

## Abstract

**Background**: Robot-assisted partial nephrectomy (RAPN) can be done using either a three-arm or four-arm configuration. However, the evidence comparing the perioperative, functional, and oncological outcomes between these two approaches is inconsistent. Therefore, we aimed to quantitatively compare the outcomes of three-arm versus four-arm RAPN. **Methods**: A comprehensive search of multiple databases, including PubMed, Embase, Scopus, Web of Science, and Cochrane, was conducted, adhering to the PRISMA guidelines. Studies comparing three-arm and four-arm RAPN were included. Continuous outcomes were assessed using mean differences (MD), and dichotomous outcomes were evaluated using risk ratios (RR). The ROBINS-I tool was used to determine the risk of bias. **Results**: Five studies that met the selection criteria were included in the final review and analysis. The pooled analyses demonstrated no significant difference in estimated blood loss, warm ischemia time, transfusion rates, overall complications, major complications, or positive surgical margins between the three-arm and four-arm RAPN. Although the initial primary analysis showed a shorter length of stay within the three-arm RAPN technique, the sensitivity analysis did not reflect this finding. **Conclusions**: The three-arm and four-arm RAPN demonstrated comparable perioperative, functional, and oncologic outcomes. As both techniques appear to be effective, the choice of configuration may be decided by the institutional resources, case complexity, and the surgeon’s preference.

## 1. Introduction

Global data suggests that kidney cancer is the fourteenth most common cancer in the world, with an incidence of 434,419 new cases and 155,702 cancer deaths in the year 2022 [1]. The increase in technological innovations and widespread adoption of radiological imaging has led to an increase in the diagnosis of RCC. In fact, 75% of newly diagnosed renal masses are incidental, asymptomatic, and smaller than 7 cm in diameter [2]. Surgical resection remains the standard treatment for kidney cancer or renal cell carcinoma (RCC). In comparison with radical nephrectomy, partial nephrectomy (PN) has the advantage of preserving long-term renal function and decreasing future cardiovascular risks, while providing comparable oncological outcomes [3,4]. Therefore, in localized RCC patients, PN is the current treatment of choice for clinical T1 tumors. Furthermore, PN has gained attention in special clinical scenarios, such as recurrent and bilateral masses [3]. Although it was initially used for small renal masses, with the introduction of robot-assisted techniques over the years, PN is being increasingly adapted even for larger and more complex masses [3,5].

In comparison to open PN, laparoscopic PN results in reduced blood loss, shorter hospital stays, and lower complication rates [6]. But certain intrinsic constraints, such as rigid instruments and 2D visualization, result in technical and ergonomic challenges during the crucial aspects of surgery, such as tumor excision and renorrhaphy [7]. These limitations became the catalyst for widespread adoption of robotic-assisted partial nephrectomy (RAPN). Currently, more than 60% of minimally invasive PN procedures are performed through a robotic approach in high-volume centers [8,9]. The evolution and increasing use of RAPN have led to improved perioperative and postoperative outcomes, such as lower rates of conversion to open or radical surgery, decreased warm ischemia time, fewer severe postoperative complications, and better preservation of postoperative renal function [4].

RAPN can be performed using either a three-arm or four-arm surgical configuration. From a technical perspective, the three-arm and four-arm robotic RAPN vary in port placement, instrument triangulation, and the degree of surgeon-controlled retraction. The four-arm configuration allows the surgeon to independently control an additional robotic instrument, and this may facilitate renal mobilization, hilar exposure, and tumor retraction, especially in anatomically challenging cases. In contrast, the three-arm RAPN relies more on the bedside assistant for retraction but offers simpler port placement, reduced instrument crowding, and potentially lower procedural costs. A schematic illustration of port placements for three-arm and four-arm RAPN, as reported across different studies included in this review, is in the Supplementary File (Supplementary Table S1) [4]. In this regard, a few studies have compared these two surgical approaches, but the findings regarding perioperative, functional, and oncological outcomes are inconsistent. Therefore, the objective of our study was to perform a systematic review and meta-analysis of the available literature comparing three-arm versus four-arm RAPN and quantitatively evaluate the various outcomes between the two surgical approaches.

## 2. Methods

Our systematic review and meta-analysis adhered to the Preferred Reporting Items for Systematic Reviews and Meta-Analyses (PRISMA) guidelines. The review is registered on PROSPERO (ID: CRD420251235763)

### 2.1. Search Strategy

A comprehensive search strategy was developed to identify all the relevant studies in the literature. The electronic databases PubMed, Embase, Scopus, Web of Science, and Cochrane were searched for articles that have been published. No restrictions were placed on the publication date. The search strategy comprised a combination of Medical Subject Headings (MeSH) and keywords such as “3 arm”, “3-arm”, “three arm”, “three arms”, “three-arm”, “triple arm”, “triple-arm”, “4 arm”, “4-arm”, “four arm”, “four arms”, “four-arm”, “quadruple arm”, “quadruple-arm”, “robot-assisted partial nephrectomy”, “robotic partial nephrectomy”, “robot assisted partial nephrectomy”, “robotic nephron sparing surgery”, “robot-assisted nephron sparing surgery”, “robot assisted nephron sparing surgery”, “RAPN”, “robot-assisted NSS”, and “robotic NSS”. Boolean operators (AND, OR) were utilized to integrate the search terms and refine the search results. (Supplementary File S1).

### 2.2. Study Selection

The titles and abstracts of the studies from our initial literature search were independently screened by two reviewers (MJRI and PP), and the studies that matched our selection criteria were included in our systematic review. A third senior reviewer (MM) was consulted for studies that had discrepancies in selection between the two reviewers. Screening of the studies and removal of duplicates was done using Rayyan (http://rayyan.qcri.org, Rayyan Systems Inc., Cambridge, MA, USA) [10]. Studies were eligible if they included patients who underwent RAPN and provided a comparison of the outcomes between the three-arm and four-arm techniques. Case reports, reviews, editorials, abstracts, and studies with obscure or incomplete information regarding the outcomes of the surgery were excluded from the review. Full texts of all the finalized articles were extracted following the initial screening. These full texts were subsequently evaluated for inclusion in the final review and analysis based on the above-mentioned selection criteria. A manual reference search was also conducted on the included articles to ensure identification of all pertinent publications.

### 2.3. Data Extraction

A template Microsoft Excel sheet was created and utilized for the data extraction and compilation. The extracted data consisted of baseline characteristics of the study (e.g., author, publication year, study period, study design, population size, demographic data, study outcomes), tumor characteristics (e.g., tumor size, location, RENAL score, histology, T-stage), and operative outcomes (e.g., estimated blood loss, operative time, warm ischemia time, blood transfusion details, length of stay, complication details, cost). All data that were extracted were cross-verified, and disparities were addressed through discussion.

### 2.4. Quality Assessment and Data Analysis

The risk of bias was assessed by two reviewers (MJRI and DKS) using the ROBINS-I tool. Mean differences (MD) with corresponding 95% confidence intervals (CIs) were used to pool continuous outcomes, and risk ratios (RR) with 95% CIs were used to pool dichotomous outcomes. All analyses were conducted using a random-effects model due to the expected clinical and methodological heterogeneity. The I^2^ statistic was used in the assessment of statistical heterogeneity. When different summary statistics were used to report the continuous outcomes, primary analyses were initially performed by including studies that reported comparable data formats. Subsequently, in the studies that reported medians with ranges or interquartile ranges, this data was converted to means and standard deviations (SD) using an established distribution-based method described by Wan et al. [11]. Sensitivity analyses were then performed to evaluate the robustness of the pooled estimates. All tests were two-sided, and a *p*-value of <0.05 was considered statistically significant. Results were demonstrated utilizing forest plots. All statistical analyses were performed using R software (version 4.5.2) with the meta package.

## 3. Results

**Figure 1:**
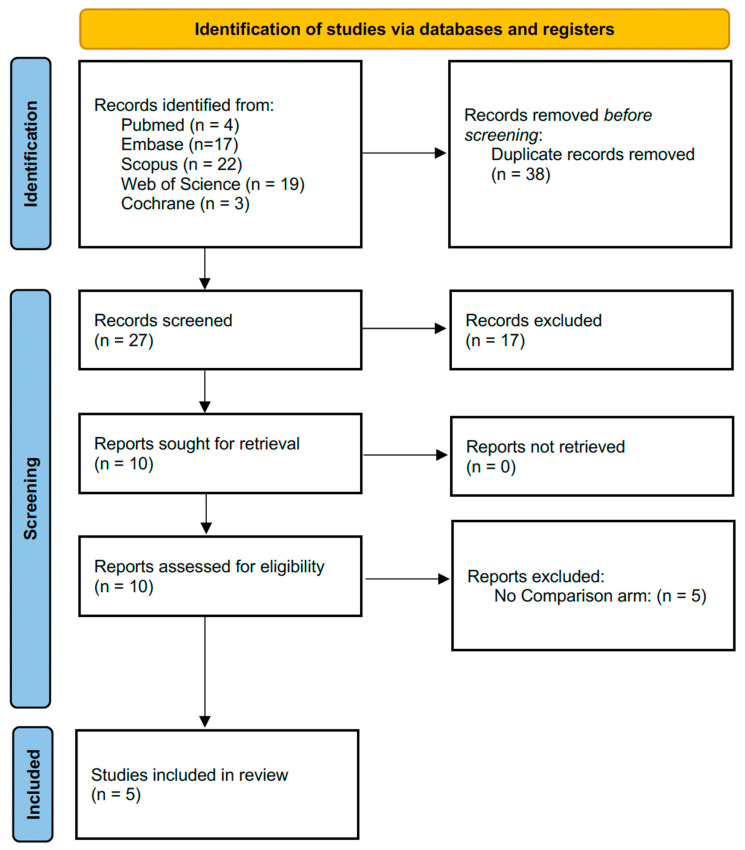
PRISMA flow diagram.

A comprehensive literature search was done through 21 December 2025. The initial search resulted in 65 articles. Following the removal of duplicates, 27 articles were assessed for eligibility. After the full-text review, 5 studies met the selection criteria and were included in the final review for qualitative and quantitative synthesis. Study selection details are summarized in the PRISMA flow diagram (Figure 1). Overall, the ROBINS-I tool demonstrated a serious risk of bias in four studies, primarily from the confounding (domain 1) due to the non-randomized allocation of the surgical technique (Supplementary Figure S1).

The baseline characteristics across the included study population are summarized in Table 1. All studies were retrospective in nature and were conducted at various sites worldwide. The total pooled cohort comprised 406 patients who underwent RAPN utilizing either a three-arm or four-arm technique. Except for one study by Lim et al., which used a retroperitoneal approach, all others employed a transperitoneal approach [4,9,12,13,14]. The Da Vinci Si robotic system was predominantly used, except in Schulze et al., where the Da Vinci Xi was also used [4]. Across studies, patients in the three-arm RAPN group were marginally older and had a higher body mass index (BMI). The primary outcomes reported by the studies included the intraoperative details, perioperative outcomes, complications, and the cost (Table 2). Length of stay reported in hours was converted to days before pooling. Tumor characteristics across different studies are summarized in Supplementary Table S2. Lim et al. and El-Asmar et al. reported a larger tumor size in the four-arm group, whereas Jhonson et al. and Schulze et al. reported a larger tumor size in the three-arm RAPN group [4,12,13,14]. All studies reported that clear-cell RCC was the predominant histological subtype, accounting for 57–77% of tumors.

**Figure 2:**
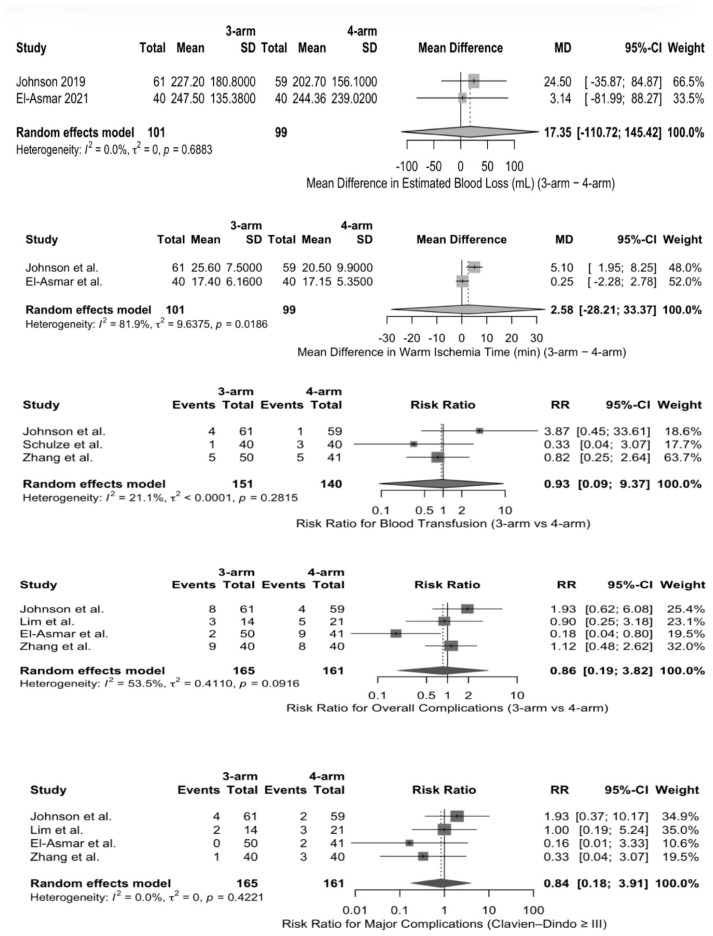
Forest plots demonstrating pooled estimates of perioperative outcomes between three-arm and four-arm RAPN (RAPN, robot-assisted partial nephrectomy; SD, standard deviation; MD, mean difference; RR, risk ratio; CI, confidence interval; I^2^, I-squared statistic; mL, milliliters; min, minutes) [4,9,12,13,14].

**Figure 3:**
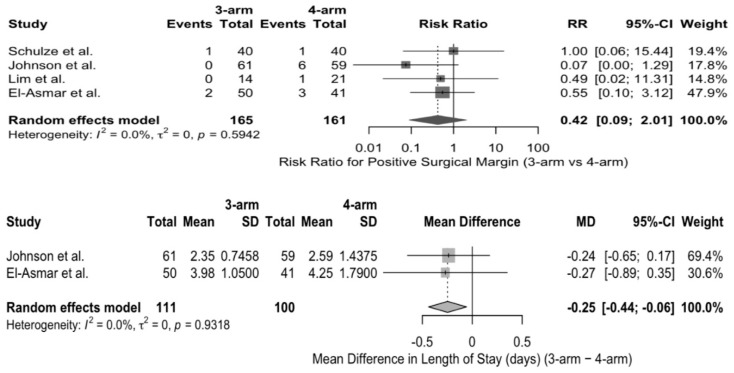
Forest plots demonstrating pooled estimates for positive surgical margins and length of stay between three-arm and four-arm RAPN (RAPN, robot-assisted partial nephrectomy; SD, standard deviation; MD, mean difference; RR, risk ratio; CI, confidence interval; I^2^, I-squared statistic) [4,12,13,14].

Estimated blood loss (EBL) was reported in milliliters (mL) in all five studies. The primary analysis of studies that reported mean ± SD did not reveal any statistically significant difference in EBL between the three-arm and four-arm RAPN. Subsequent sensitivity analysis by incorporating studies with converted median-based estimates also demonstrated similar results, with no significant difference observed between the two groups. Similarly, there was no significant difference in the pooled analysis of warm ischemia time (WIT) between the three-arm and four-arm RAPN. Sensitivity analysis by including studies with converted median-based estimates also did not alter the pooled effect. But the primary analysis of studies reporting the length of stay (LOS) in mean ± standard deviation, three-arm RAPN was associated with lower LOS in comparison with four-arm RAPN (MD: −0.25 days, 95% CI: −0.44 to −0.06; *p* = 0.039), with no observed heterogeneity (I^2^ = 0%). However, when studies with converted median-based estimates were also included during sensitivity analysis, the difference in LOS between the two patient groups was no longer statistically significant (MD: −0.21 days, 95% CI: −1.27 to 0.86; *p* = 0.58), and substantial heterogeneity was observed (I^2^ = 64.2%). (Supplementary Figure S2) Pooled analysis of various studies did not reveal any statistically significant difference in terms of perioperative blood transfusion, overall complications, major complications (Clavien–Dindo ≥ III), or positive surgical margins (Figure 2 and Figure 3).

## 4. Discussion

In this systematic review and meta-analysis that compared the three-arm and four-arm RAPN, it was found that perioperative, functional, and oncologic outcomes were comparable between the two surgical approaches. The pooled analyses did not reveal any significant difference in the estimated blood loss, warm ischemia time, transfusion rates, overall complications, major complications, and positive surgical margin rates between the two groups. Although the length of stay was marginally less in the three-arm RAPN approach during the primary analysis, subsequent sensitivity analysis did not sustain this finding.

As contemporary urological practice focuses on maintaining oncological control while preserving renal function and limiting perioperative morbidity, RAPN has emerged as an important option. WIT is a recognized factor that is known to impact the renal functional outcome and is also part of composite quality metrics such as “trifecta” [15]. While individual studies reported variability in the technique associated with longer WIT, the pooled analysis did not demonstrate any statistically significant difference in WIT between the three-arm and four-arm RAPN techniques. The trifecta in RAPN is a composite outcome measure that includes minimal renal functional decrease, negative surgical margins, and absence of major postoperative complications following the procedure [15,16]. Very few positive surgical margins were recorded across the included studies, and no statistically significant difference was observed in the pooled analysis. Variability in overall and major complication rates (Clavien–Dindo grade ≥ III) was observed across studies, with no statistically significant difference identified in the pooled analysis. The absence of a significant difference in the perioperative outcomes suggests that the three-arm RAPN approach achieves comparable results to the four-arm approach. Outcomes that are considered to be surrogates of surgical complexity, such as EBL and WIT, were similar in both groups. These findings suggest that the surgical familiarity, expertise, and case selection could play a more important role than the robotic configuration alone in determining the perioperative outcomes and the trifecta.

The functional outcomes also did not differ between the three-arm and four-arm RAPN approaches. Johnson et al. reported a mean change (SD) in the serum creatinine to be −0.04 (0.3) in the three-arm RAPN and 0.01 (0.4) in the four-arm RAPN (*p* = 0.503) [12]. Similarly, El-asmar et al. showed the mean (SD) GFR difference to be −10.25 (10.09) and −8.41 (9.22) in the three-arm and four-arm RAPN, respectively [14]. Regarding the renal functional outcomes, available data were limited and derived from a small number of studies with heterogeneous definitions and follow-up intervals. Therefore, quantitative synthesis was not feasible, and any conclusions regarding functional equivalence should be considered preliminary. The difference in cost across the techniques was reported only in two studies. Schulze et al. reported that the total amount saved with the three-arm RAPN was USD $413.00 for each surgery. They commonly used the ProGrasp^®^ (Intuitive Surgical) grasper as the fourth arm during the dissection of the kidney hilum and renorrhaphy. The cost difference was calculated after considering the cost of the fourth arm, disposable adjuncts, and sterile covers [4]. Similarly, Zhang et al. reported that the total costs for the three-arm RAPN were significantly lower than the four-arm approach (CNY: 68,406.7 vs. CNY: 76,922.5; *p* = 0.006) [9]. These cost savings may have higher clinical relevance in resource-limited settings, where access to robotic equipment and financial constraints can have an impact on the surgical strategy and adoption of technologies.

The primary analysis of studies reporting LOS in mean ± SD demonstrated decreased duration in the three-arm RAPN group. But, this result was not sustained during subsequent sensitivity analysis, incorporating other studies requiring conversion from median-based reporting of data. This pattern requires recognition, as LOS is heavily influenced by institutional practices and is sensitive to the differences in reporting format across studies. To overcome such limitations of heterogeneous data reporting and summary statistics in LOS and other outcomes, a restricted primary analysis was first performed, followed by sensitivity analyses incorporating converted estimates. While the pooled analyses did not demonstrate statistically significant differences between configurations across key outcomes such as EBL, WIT, complications, and positive surgical margins, the absence of detected differences does not necessarily imply equivalence, particularly in the context of non-randomized study designs and residual confounding. Beyond statistical heterogeneity, significant clinical heterogeneity exists across the included studies. Factors such as surgeon experience, institutional volume, docking strategy, bedside assistant expertise, and learning curve effects may influence perioperative and oncologic outcomes and potentially overshadow the isolated impact of robotic arm configuration. Variability in these contextual factors may partially explain the absence of consistent differences observed between the three-arm and four-arm RAPN. Consequently, arm configuration should be understood as one component within a broader operative ecosystem rather than as an independent determinant of outcomes. Furthermore, tumor size, RENAL nephrometry scores, and reporting of the overall case complexity varied across the studies. Therefore, stratified analyses based on tumor complexity (for instance, RENAL score ≥7 versus <7) were not feasible due to inconsistent reporting and the lack of patient-level data across studies. So, it remains unclear whether one technique could be more advantageous over the other in more complex or anatomically challenging tumors. Therefore, future studies should incorporate standardized complexity metrics and stratified outcome reporting.

A few limitations must be acknowledged. First, all studies included in the review were predominantly retrospective in nature, which could introduce inherent selection bias and unmeasured confounding. Accordingly, ROBINS-I assessment demonstrated a moderate to serious risk of bias across all the studies. Second, the studies had some variations in outcome definitions and the reporting format of the data. This required data transformation for some continuous outcomes that could introduce estimation uncertainty despite the use of standard methods and sensitivity analyses. Third, factors that can impact the outcomes of the surgery, such as surgeon experience, institutional volume, and institutional practices, could not be uniformly assessed across the studies. Fourth, all studies had modest sample sizes and were derived from a single institution, which limits their generalizability. Fifth, due to heterogeneity in the reporting and timing of postoperative renal functional outcomes, a formal quantitative meta-analysis of functional endpoints was not feasible. This further highlights the need for future prospective studies with standardized reporting of renal functional outcomes. Sixth, although the pooled analyses demonstrated no statistically significant differences between the two approaches across key perioperative and oncologic outcomes, the overall certainty of evidence should be interpreted with caution. All studies were retrospective in nature, and the ROBINS-I assessment identified moderate to serious risk of bias. As a result, the certainty of evidence supporting outcomes such as estimated blood loss, warm ischemia time, complication rates, and positive surgical margins is best considered low to moderate. These findings should therefore be interpreted as an absence of detected differences rather than definitive equivalence between techniques. This highlights the need for well-designed prospective or randomized studies. Finally, as the long-term outcomes, including functional and oncological outcomes, were not consistently reported, assessment beyond the perioperative period was limited. Therefore, future studies should focus on large, multicenter, prospectively maintained datasets with uniform definitions of outcomes, standard practice, and an extended follow-up. This would ensure the comprehensive evaluation of both short-term and long-term outcomes.

## 5. Conclusions

This systematic review and meta-analysis found that the perioperative, functional, or oncologic outcomes did not have any statistically significant difference between three-arm and four-arm techniques of RAPN across key outcomes such as EBL, WIT, complications, and positive surgical margins. Overall, both techniques appear to be equally effective, and the choice should depend on the surgeon’s preference, institutional resources, and case complexity.

## Figures and Tables

**Table 1:** Baseline characteristics of the patient population across included studies.

Study (Year)	Country	Study Design	Study Period	Study Population		Approach (TP/RP)	Robot Platform	No of Surgeons	Age (Years)		BMI (kg/m^2^)		Primary Outcomes Reported
				3-Arm	4-Arm				3-Arm	4-Arm	3-Arm	4-Arm	
Schulze et al. (2022) [4]	Brazil	Retrospective	2016–2020	40	40	TP	Da Vinci Si and Xi	2	57.48 (20–75)	56.63 (34–77)	NR		Intraoperative details, Perioperative outcomes, complications, cost
Johnson et al. (2019) [12]	USA	Retrospective	2016–2017	61	59	TP	Da Vinci Si	5	61 (47–68)	60 (54–67)	30.7 (25.1–30.7)	29.7 (25.8–33.2)	Intraoperative details, Perioperative outcomes, complications, cost
Lim et al. (2018) [13]	Taiwan	Retrospective	2012	14	21	RP	Da Vinci Si	1	61.5 (35–74)	46 (32–77)	26.9 (17.4–33.9)	26.2 (20.4–32.7)	Intraoperative details, Perioperative outcomes, complications
Zhang et al. (2025) [9]	China	Retrospective, PSM	2021–2023	50	41	TP	Da Vinci Si	1	56.0 (49.8–60.3)	56.0 (48.0–63.0)	24.7 (±3.2)	23.9 (±2.6)	Intraoperative details, Perioperative outcomes, complications, cost
El-Asmar et al. (2021) [14]	Lebanon	Retrospective	2013–2017	40	40	TP	Si	NR	57 *		30.3 *		Intraoperative details, Perioperative outcomes, complications

PSM: Propensity Score Matched; TP: Transperitoneal; RP: Retroperitoneal; BMI: Body mass index; NR: Not recorded; * Age/BMI reported overall, not stratified by arm.

**Table 2:** Perioperative and cost-related outcomes across the included studies.

Study	EBL (mL)		WIT (Min)		Op Time (Min)		Transfusion		Clavien ≥ III n (%)		Complication n (%)		Positive Margin (%)		LOS		Cost	
	3-Arm	4-Arm	3-Arm	4-Arm	3-Arm	4-Arm	3-Arm	4-Arm	3-Arm	4-Arm	3-Arm	4-Arm	3-Arm	4-Arm	3-Arm	4-Arm	3-Arm	4-Arm
Schulze et al. [4]	Avg: 221 (30–800)	Avg: 325 (20–2250)	Mean: 16.25 (0–35)	Mean: 21.78 (0–50)	Avg: 81 (29–215) *	Avg: 91 (40–180) *	2.5%	7.5%	NR	NR	NR	NR	2.5%	2.5%	NR	NR	Lower by US $413	—
Johnson et al. [12]	227.2 ± 180.8	202.7 ± 156.1	25.6 ± 7.5	20.5 ± 9.9	176.6 ± 34.1	184.3 ± 46.9	4 (6%)	1 (2%)	4 (7%)	2 (3%)	8 (13%)	4 (7%)	0	6 (10%)	56.4 ± 17.9 h	62.1 ± 34.5 h	—	Higher (not quantified)
Lim et al. [13]	125 (50–1000)	150 (50–1700)	23 (17–28)	17 (5–34)	139 (80–235) *	110 (40–218) *	NR	NR	2 (14.3%)	3 (14.3%)	3 (21.4%)	5 (23.9%)	0	1 (4.8%)	4 (2–8) d	4 (2–18) d	—	—
Zhang et al. [9]	150 (100, 300)	200 (100, 362.5)	28 (21.8, 33.3)	30 (25.8, 39.3)	120 (100, 135)	146.5 (101.5, 177.8)	5 (13.2%)	5 (13.2%)	1 (2.6%)	3 (7.9%)	9 (23.7%)	8 (21.1%)	NR	NR	6 (5, 7) d	5 (5, 7) d	—	+8516 CNY
El-Asmar et al. [14]	247.50 ± 135.38	244.36 ± 239.02	17.40 ± 6.16	17.15 ± 5.35	NR	NR	NR	NR	0	2 (5%)	2 (5%)	9 (22.5%)	2 (5%)	3 (7.5%)	3.98 ± 1.05 d	4.25 ± 1.79 d	—	—

* Console time. EBL: Estimated Blood Loss; mL: Milliliters; Min: minutes; WIT: Warm Ischemia Time; Op time: Operative Time. Clavien: Clavien–Dindo Classification; LOS: Length of Stay; Avg: Average; NR: Not Recorded; h: Hours; d: Days (LOS reported in hours or days as per original study); US $: United States Dollars; CNY: Chinese Yuan.

## Data Availability

Data are available from the corresponding author upon reasonable request.

## Supplementary Materials

The following supporting information can be downloaded at: https://www.mdpi.com/article/10.3390/jcm15031222/s1, Figure S1: Risk of Bias Assessment of Included Studies Using the ROBINS-I tool; Figure S2: Sensitivity analyses comparing perioperative outcomes (estimated blood loss, warm ischemia time, length of stay) between the three-arm and four-arm RAPN; Table S1: Schematic representation of port placements in three-arm and four-arm RAPN in different studies; Table S2: Tumor-specific characteristics across the included studies; Supplementary S1: Search String; File S1: PRISMA 2020 checklist [17].
