# Supplementary material for: Three-Arm Versus Four-Arm Configurations in Robot-Assisted Partial Nephrectomy: A Systematic Review and Meta-Analysis
Source: J Clin Med. 2026 Feb 4;15(3):1222. doi: 10.3390/jcm15031222 (PMC12897796; doi:10.3390/jcm15031222)
Supplement: Supplementary file 1 [file jcm-15-01222-s001.zip › jcm-4108497-supplementary.pdf]

Supplementary Figure 1: Risk of Bias Assessment of Included Studies Using the ROBINS-I tool

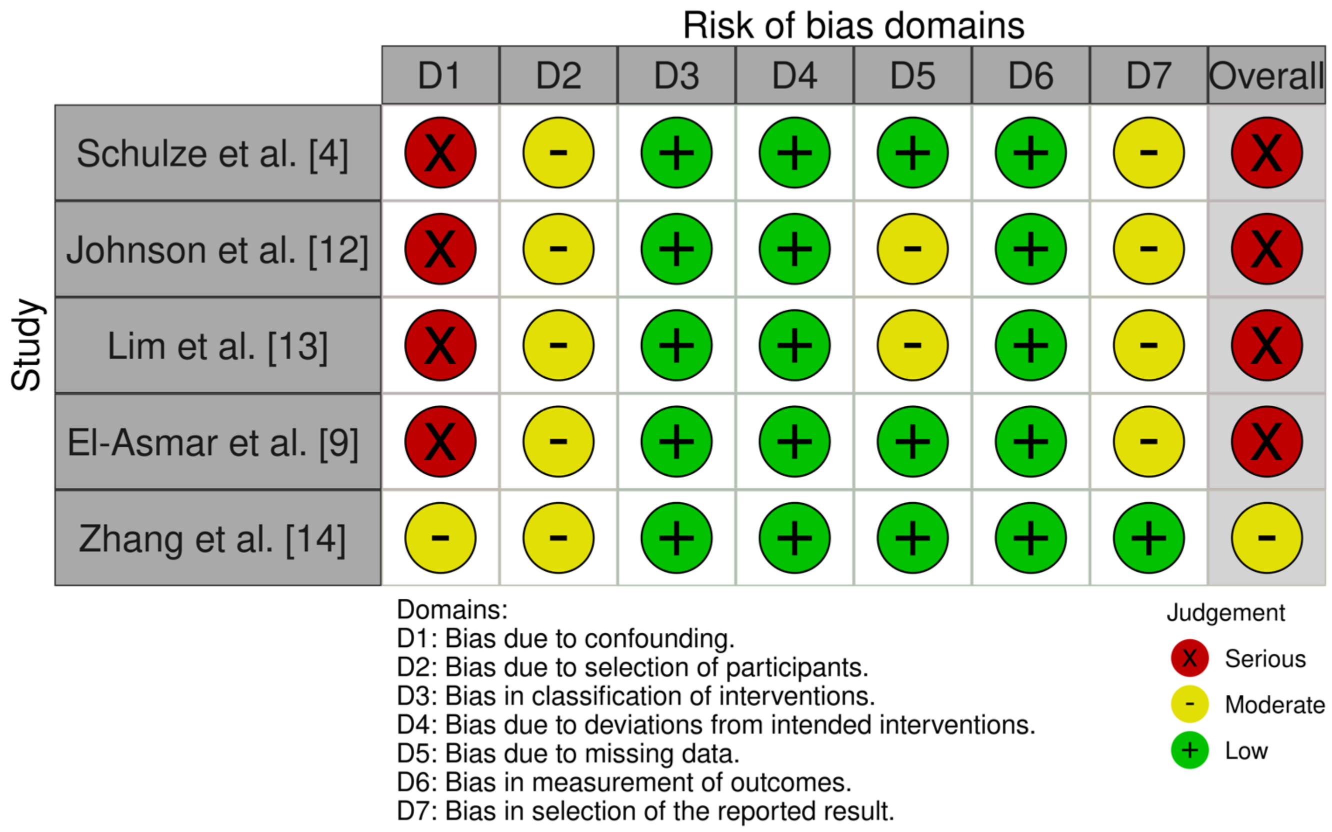

Supplementary Table 1: Schematic representation of port placements in three-arm and four-arm RAPN in different studies

Study	Port placement
Schulze et al. [4]	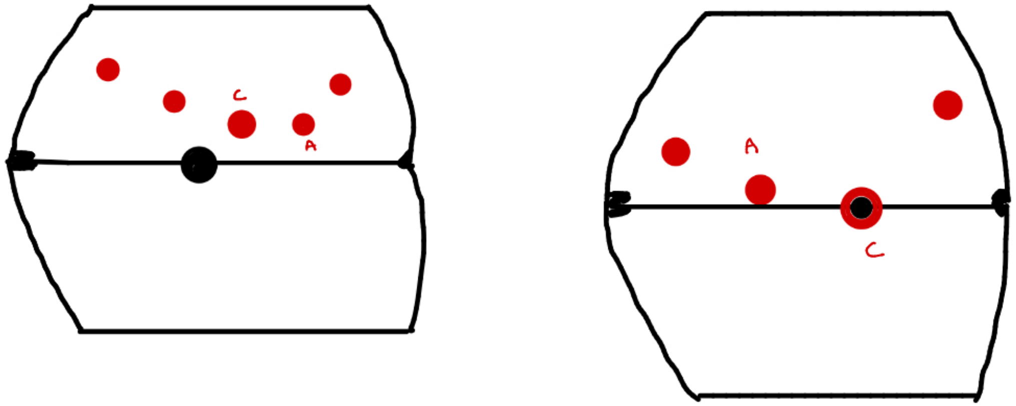<br>Port placement of the right side RAPN. (C – Camera port; A – Assistant port)<br>Four-arm RAPN: Camera port placed medially to the umbilicus (C). Three-arm RAPN: Camera port placed in the umbilicus
Lim et al. [13]	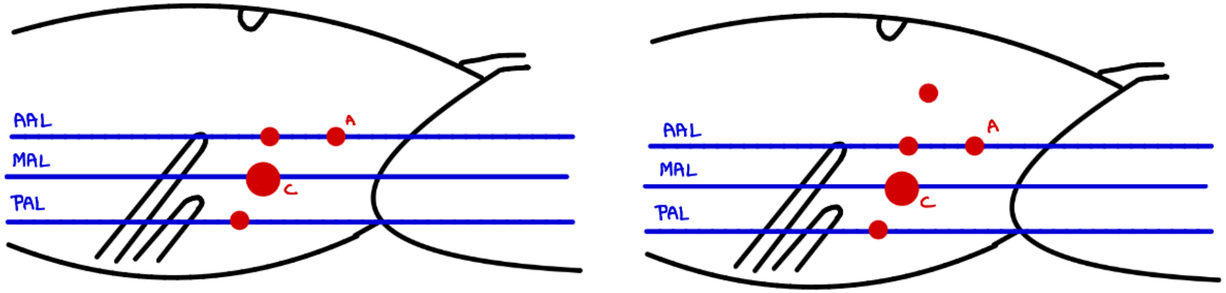<br>Port placement of the right side RAPN. (C – Camera port; A – Assistant port; AAL – Anterior Axillary Line; MAL – Mid Axillar Line; PAL – Posterior Axillary Line)

Supplementary Table 2: Tumor-specific characteristics across the included studies

Study	Tumor Size (cm)		RENAL Score		Tumor Side (R/L)		Tumor Location		Histology		T-stage	
	3-arm	4-arm	3-arm	4-arm	3-arm	4-arm	3-arm	4-arm	3-arm	4-arm	3-arm	4-arm
Schulze et al. [4]	3.82 (1.3–10)	3.49 (1–8.5)	4–6: 47.5%<br>7–9: 22.5%<br>≥10: 30%	4–6: 50%<br>7–9: 25%<br>≥10: 25%	R: 45%<br>L: 55%,	R: 52.5%<br>L: 47.5%	NR	NR	Cc: 72.5%<br>Pap*: 5%<br>AML: 10%<br>Onc: 2.5%<br>Others:10%	Cc: 60%<br>Pap**: 5%<br>AML: 10%<br>Onc: 5%<br>Chromo: 10%<br>Others: 10%	T1a: 80%<br>T1b: 5%<br>T2: 12.5%<br>T3: 2.5%	T1a: 70%<br>T1b: 22.5%<br>T2: 7.5%<br>T3: 0%
Johnson et al. [12]	3.4 ± 1.1	3.3 ± 1.2	7 (6–8)	6 (5–8)	R: 54%<br>L: 46%	R: 39%<br>L: 61%	NR	NR	Cc: 77%<br>Pap: 2%<br>Chromo: 10%<br>Onc: 2%<br>Benign: 15%	Cc: 70%<br>Pap: 5%<br>Chromo: 10%<br>Onc: 8%<br>Benign: 5%	T1a: 69%<br>T1b: 20%<br>T3a: 10%	T1a: 74%<br>T1b: 23%<br>T3a: 4%
Lim et al. [13]	2.3 (1–6.7)	3.5 (1–6.9)	Low: 64.29%, Int: 35.71%	Low: 33.33%, Int: 47.63%, High: 19.05%	R: 42.9%<br>L: 57.1%	R: 42.9%<br>L: 57.1%	Ant: 50%<br>Post: 28.57%	Ant: 23.81%<br>Post: 38.10%	Cc: 57.1%<br>Pap: 7.1%<br>Chromo: 21.4%<br>Benign: 14.3%	Cc: 66.7%<br>Pap: 9.5%<br>Chromo: 4.8%<br>Benign: 19%	NR	NR
El-Asmar et al. [9]	3.14 ± 1.19	3.44 ± 1.60	Low: 57.5%, Int:	Low: 36.1%, Int: 58.3%,	NR	NR	NR	NR	Cc: 60%<br>Pap1: 17.5%<br>Pap2: 2.5%	Cc: 57.5%<br>Pap1: 5%<br>Pap2: 5%	NR	NR

			37.5%, High: 5%	High: 5.6 %					Onc: 7.5%<br>AML: 10%<br>Others: 2.5%	Onc: 17.5%<br>Others: 15%		
Zhang et al. [14]	NR	NR	7.0 (6–9)	7.0 (7–9)	R: 52%<br>L: 48%	R: 63.4%<br>L: 36.6%	NR	NR	NR	NR	T1a: 86%<br>T1b: 6%<br>T2a: 6%<br>T2b: 2%	T1a: 75.6%<br>T1b: 19.5%<br>T2a: 4.9%<br>T2b: 0%

R: Right; L: Left; Ant: Anterior; Post: Posterior; Cc: Clear cell renal cell carcinoma; Pap: Papillary renal cell carcinoma; Pap1: Papillary type 1; Pap2: Papillary type 2; Chromo: Chromophobe renal cell carcinoma; Onc: Oncocytoma; AML: Angiomyolipoma; NR: Not reported

Supplementary Figure 2: Sensitivity analyses comparing perioperative outcomes (estimated blood loss, warm ischemia time, length of stay) between the three-arm and four-arm RAPN

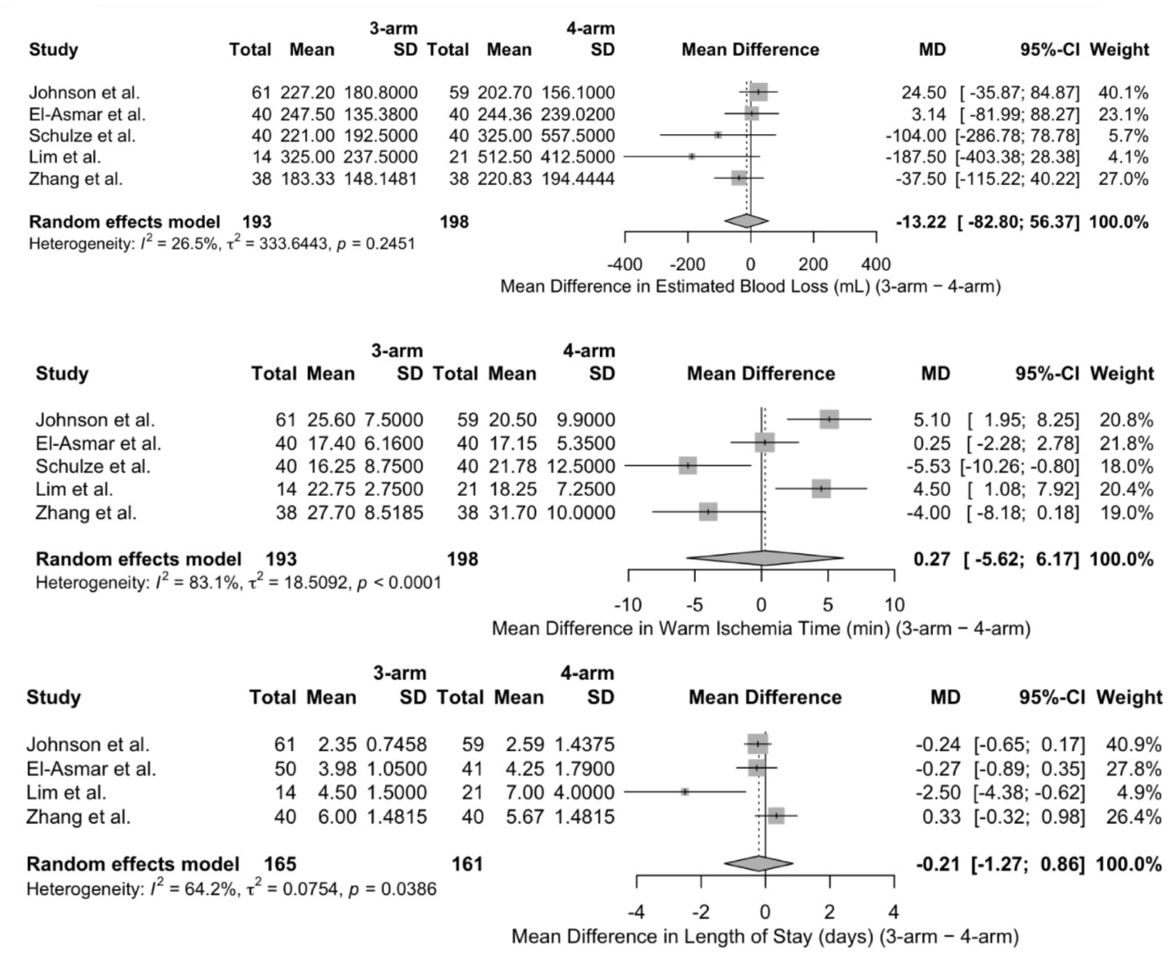

Search String:

PubMed: 4 Articles

Search number	Query	Filters	Search Details	Results
2	(("3 arm" OR "3-arm" OR "three arm" OR "three arms" OR "three-arm" OR "triple arm" OR "triple-arm") OR ("4 arm" OR "4-arm" OR "four arm" OR "four arms" OR "four-arm" OR "quadruple arm" OR "quadruple-arm")) AND ("robot-assisted partial nephrectomy" OR "robotic partial nephrectomy" OR "robot assisted partial nephrectomy" OR "robotic nephron sparing surgery" OR "robot-assisted nephron sparing surgery" OR "robot assisted nephron sparing surgery" OR "RAPN" OR "robot-assisted NSS" OR "robotic NSS")	Case Reports, Classical Article, Clinical Study, Clinical Trial, Clinical Trial, Phase I, Clinical Trial, Phase II, Clinical Trial, Phase III, Clinical Trial, Phase IV, Controlled Clinical Trial, Evaluation Study, Multicenter Study, Observational Study, Randomized Controlled Trial, Technical Report	(("3-arm"[All Fields] OR "3-arm"[All Fields] OR "three-arm"[All Fields] OR "three arms"[All Fields] OR "three-arm"[All Fields] OR "triple-arm"[All Fields] OR "triple-arm"[All Fields] OR ("4-arm"[All Fields] OR "4-arm"[All Fields] OR "four-arm"[All Fields] OR "four arms"[All Fields] OR "four-arm"[All Fields] OR "quadruple-arm"[All Fields] OR "quadruple-arm"[All Fields])) AND ("robot-assisted partial nephrectomy"[All Fields] OR "robotic partial nephrectomy"[All Fields] OR "robot-assisted partial nephrectomy"[All Fields] OR "robotic nephron sparing surgery"[All Fields] OR "robot-assisted nephron sparing surgery"[All Fields] OR "robot-assisted nephron sparing surgery"[All Fields] OR "RAPN"[All Fields] OR "robot-assisted NSS"[All Fields] OR "robotic NSS"[All Fields])) AND (casereports[Filter] OR classicalarticle[Filter] OR clinicalstudy[Filter] OR clinicaltrial[Filter] OR clinicaltrialphasei[Filter] OR clinicaltrialphaseii[Filter] OR clinicaltrialphaseiii[Filter] OR clinicaltrialphaseiv[Filter] OR controlledclinicaltrial[Filter] OR evaluationstudy[Filter] OR multicenterstudy[Filter] OR observationalstudy[Filter] OR randomizedcontrolledtrial[Filter] OR technicalreport[Filter])	4
1	(("3 arm" OR "3-arm" OR "three arm" OR "three arms" OR "three-arm" OR "triple arm" OR "triple-arm") OR ("4 arm" OR "4-arm" OR "four arm" OR "four arms" OR		("3-arm"[All Fields] OR "3-arm"[All Fields] OR "three-arm"[All Fields] OR "three arms"[All Fields] OR "three-arm"[All Fields] OR "triple-arm"[All	22

	"four-arm" OR "quadruple arm" OR "quadruple-arm")) AND ("robot-assisted partial nephrectomy" OR "robotic partial nephrectomy" OR "robot assisted partial nephrectomy" OR "robotic nephron sparing surgery" OR "robot-assisted nephron sparing surgery" OR "robot assisted nephron sparing surgery" OR "RAPN" OR "robot-assisted NSS" OR "robotic NSS")	Fields] OR "triple-arm"[All Fields] OR ("4-arm"[All Fields] OR "4-arm"[All Fields] OR "four-arm"[All Fields] OR "four arms"[All Fields] OR "four-arm"[All Fields] OR "quadruple-arm"[All Fields] OR "quadruple-arm"[All Fields])) AND ("robot-assisted partial nephrectomy"[All Fields] OR "robotic partial nephrectomy"[All Fields] OR "robot-assisted partial nephrectomy"[All Fields] OR "robotic nephron sparing surgery"[All Fields] OR "robot-assisted nephron sparing surgery"[All Fields] OR "robot-assisted nephron sparing surgery"[All Fields] OR "RAPN"[All Fields] OR "robot-assisted NSS"[All Fields] OR "robotic NSS"[All Fields])	

EMBASE: 17 Articles

#2 AND **'article'**/it 17

#1 ('3 arm' OR '3-arm' OR 'three arm' OR 'three arms' OR 'three-arm' OR 'triple arm' OR 'triple-arm' OR '4 arm' OR '4-arm' OR 'four arm' OR 'four arms' OR 'four-arm' OR 'quadruple arm' OR 'quadruple-arm') AND ('robot-assisted partial nephrectomy' OR 'robotic partial nephrectomy'/exp OR 'robotic partial nephrectomy' OR 'robot assisted partial nephrectomy'/exp OR 'robot assisted partial nephrectomy' OR 'robotic nephron sparing surgery' OR 'robot-assisted nephron sparing surgery' OR 'robot assisted nephron sparing surgery' OR rapn OR 'robot-assisted nss' OR 'robotic nss') 75

Scopus: 22 Articles

(TITLE-ABS-KEY ("3-arm" OR "3 arm" OR "three-arm" OR "three arm" OR "three arms" OR "triple arm" OR "triple-arm" OR "4-arm" OR "4 arm" OR "four-arm" OR "four arm" OR "four arms" OR "quadruple arm" OR "quadruple-arm")) AND (TITLE-ABS-KEY ("robot-assisted partial nephrectomy" OR "robotic partial nephrectomy" OR "robot assisted partial nephrectomy" OR "robotic nephron sparing surgery" OR "robot-assisted nephron sparing surgery" OR "robot assisted nephron sparing surgery" OR RAPN OR "robot-assisted NSS" OR "robotic NSS")) AND (LIMIT-TO (DOCTYPE , "ar")) AND (LIMIT-TO (LANGUAGE , "English"))

WoS: 19 Articles

TS=(("3-arm" OR "3 arm" OR "three-arm" OR "three arm" OR "three arms" OR "triple arm" OR "triple-arm" OR "4-arm" OR "4 arm" OR "four-arm" OR "four arm" OR "four arms" OR "quadruple arm" OR "quadruple-arm")) AND TS=(("robot-assisted partial nephrectomy" OR "robotic partial nephrectomy" OR "robot assisted partial nephrectomy" OR "robotic nephron sparing surgery" OR "robot-assisted nephron sparing surgery" OR "robot assisted nephron sparing surgery" OR RAPN OR "robot-assisted NSS" OR "robotic NSS")) and Article (Document Types) and English (Languages)

Cochrane: 3 Articles

#1 - (("3-arm" OR "3 arm" OR "three-arm" OR "three arm" OR "three arms" OR "triple arm" OR "triple-arm" OR "4-arm" OR "4 arm" OR "four-arm" OR "four arm" OR "four arms" OR "quadruple arm" OR "quadruple-arm")) AND (("robot-assisted partial nephrectomy" OR "robotic partial nephrectomy" OR "robot assisted partial nephrectomy" OR "robotic nephron sparing surgery" OR "robot-assisted nephron sparing surgery" OR "robot assisted nephron sparing surgery" OR RAPN OR "robot-assisted NSS" OR "robotic NSS"))

Total No. of Articles from 5 Databases: 65 Articles
Duplicates Removed: 38 Articles
No. of Articles for Title and Abstract Screening: 27 Articles
No. of Articles Excluded: 17
No. of Articles for Full-text Screening: 10
No. of Articles Excluded: 5
No. of Articles included in the Review: 5
